# Antimony in Polyethylene Terephthalate-Bottled Beverages: The Migration Puzzle

**DOI:** 10.3390/molecules28207166

**Published:** 2023-10-19

**Authors:** Sergio Carneado, José Fermín López-Sánchez, Ángeles Sahuquillo

**Affiliations:** Analytical Chemistry Section, Faculty of Chemistry, Universitat de Barcelona, Martí i Franquès 1-11, 08028 Barcelona, Spain; sergiocarneado@gmail.com (S.C.); fermin.lopez@ub.edu (J.F.L.-S.)

**Keywords:** antimony migration, antimony speciation, juice, water, polyethylene terephthalate bottle, cross-migration experiments

## Abstract

A novel strategy to assess the main variables that potentially affect the migration of antimony from PET bottles to beverages, including mineral waters and juices, is herein proposed. In a preliminary step, an LC-ICP-MS method previously used for water analysis was optimized to correct identify Sb species present in the studied matrices using HRMS. Subsequently, the influence of temperature and storage time up to 30 days on Sb migration from PET bottles into peach and pineapple juices of the same brand was studied. Storing PET bottled drinks at elevated temperatures (i.e., in a hot car or in summer) can cause antimony migration to exceed the limits allowed in the EU or USA. Because the behavior observed differed from the results reported for Sb migration in mineral waters, a second approach was proposed: three mineral water and two juice samples were kept in different PET containers and stored at an elevated temperature (up to 60 °C) to understand the role of the PET type and matrix simultaneously. This study demonstrated that both matrix characteristics and type of PET bottle greatly influence antimony leaching, highlighting the need to consider these variables together when conducting migration experiments. The obtained results can be helpful for developing future legislation concerning migration of pollutants from packing to food commodities.

## 1. Introduction

The use of polyethylene terephthalate (PET) for food packaging is growing faster than that of any other plastic. Antimony trioxide (Sb_2_O_3_) is a common substance used in the manufacture of PET and is the most important catalyst used in the process. One of the proposed mechanisms for the incorporation of antimony into PET during the polycondensation process involves the conversion of Sb_2_O_3_ into Sb-glycolate in different forms [1]. However, X-ray absorption fine-structure spectroscopy of PET material showed that Sb(III) can be partially oxidized (up to 50%) to Sb(V), which may be more soluble than trivalent species [2]. Thus, according to the different proposals suggested by these authors, both Sb(III) and Sb(V) forms could be present in the PET surface and be in direct contact with packaged foodstuffs, even though polymer characteristics and PET packaging production can also play a role on antimony diffusivity [3].

The toxicity of antimony depends on its chemical form and oxidation state. Between the inorganic forms, Sb(III) is ten times more toxic than Sb(V) [4,5], whereas the organic antimony compounds are much less toxic. Thus, the United States Environmental Protection Agency (USEPA) [6] and the European Union (EU) [7] consider Sb a priority pollutant.

Total antimony contents from 100 to 400 mg kg^−1^ have been reported in PET containers irrespective of the commercial brand tested. In most cases, they are destined to the storage of mineral water, whereas in some works, no specification of the type of beverage or foodstuff to be stored is described [2,8,9,10,11,12,13,14,15,16]. Although these values are significantly high, the Sb concentrations in the corresponding bottled water samples are within the recommended values for drinking water. All the studies cited concluded that the presence of Sb in the bottled water was due to its migration from PET, as water obtained from a fresh source or bore or stored in plastic of other types, such as polypropylene (PP), low-density and high-density polyethylene (LDPE, HDPE), polystyrene (PS), or polycarbonate (PC), did not suffer from Sb contamination [8,9,17,18,19].

Antimony migration studies from PET into food products can provide useful information about the potential risk associated with its presence under different storage conditions. Several physicochemical factors, such as storage time, temperature, light irradiation, PET color, and matrix characteristics (e.g., pH and salt content), can influence Sb migration from PET containers to water samples [8,9,10,11,13,15,17,18,19,20,21,22,23,24,25]. However, there is a wide variability in the effects of these factors on Sb migration [3], and the predominant role of the PET type or the water matrix is not clear [3,26,27].

Much less information has been reported about the total antimony contents in other beverages bottled in PET, which have more complex matrices that include considerable amounts of salts or organic acids, such as soft drinks, red wine, and fruit juices. Although some soft drinks present higher Sb concentrations than water samples [13], in some cases, the final concentrations have been reported to be within the maximum European limit for drinking water [13,28,29], while antimony concentrations in some fruit juices exceed the limit [30]. Hansen and co-workers mentioned that Sb concentrations can be influenced by the carbohydrate content of the juice and the number of days beyond the expiry date.

Few studies identify, quantify, and establish the behavior and fate of Sb species migrating from PET into beverages or other liquid foods. The presence of different Sb(III) or Sb(V) coordination complexes has been confirmed in fruit juices, yogurt, vinegars, and spirits stored in PET, using liquid chromatography–inductively coupled plasma–mass spectrometry (LC-ICP-MS) and liquid chromatography–electrospray–mass spectrometry (LC-ES-MS). The chemical forms found were uncomplexed Sb(V) as Sb(OH)_6_^-^ or highly stable complexes with different coordination numbers between antimony and either citrate, adenosine and lactate [31,32], acetate [33], and pyruvic acid and hydroxy ethane sulfonic anions [34]. The few migration studies were performed on carbonated and non-carbonated juices bottled in PET and in spirits [34,35], where the storage effect at 50 or 60 °C was assessed for 15 days. The increase in antimony concentration observed in the carbonated samples was attributed to the increase in the migration of Sb(III), whereas the increase observed in spirits was attributed to a simultaneous release of antimony from PET and a conversion of the Sb species.

Welle and Franz [12] developed a mathematical model to predict migration of antimony into beverages with increasing temperature and time by using only the experimental data obtained from migration assays with food simulants and considering different variables including total Sb concentration in the PET, bottle volume, wall thickness, energy, and diffusion coefficients. They found that the calculated antimony concentrations were in good agreement with the data from the literature, pointing out the role of the PET container in migration.

According to the current state of this topic, the objective of this study was to investigate the factors affecting the migration of antimony from PET to beverages considering the plastic type, kinetic factors (time and temperature), and drink matrix (water and juices). For this purpose, a new methodological approach was tested: Firstly, the effect of storing temperature and time were assessed in pineapple and peach juices bottled in PET, extending a previous study on PET bottled water samples [15]. Storage temperatures from 4 to 60 °C and storage times up to 60 days were considered. Secondly, to understand the roles of both PET-type and matrix, a cross-migration experiment exchanging matrices and containers was carried out. Moreover, as there is little information on antimony speciation, the determination of total Sb and its species was performed in all batches of experiments, covering both identification of Sb species and quantification.

## 2. Results

### 2.1. Determination of Total Sb in Juices

The results for total Sb content in the seven juices (apple, grape, pear, pineapple, peach, orange, and tomato) are summarized in Table 1. 

As can be seen, Sb concentration levels were low in all cases but quantifiable (higher than the limit of quantification 0.02 μg·L^−1^).

### 2.2. Sb Speciation in Juices

With the optimized chromatographic method, the seven juice matrices were quantified. The concentrations of the uncomplexed Sb(V) were near the limit of detection (0.01 µg L^−1^) in all samples. The concentrations of Sb(III) ranged between 0.12 and 0.42 µg L^−1^, with a mean concentration of 0.29 µg L^−1^ and relative standard deviation (RSD) values from 0.7 to 10% (*n* = 3). 

### 2.3. Migration Experiments

The effect of different storage temperatures on the migration of total Sb and Sb speciation with time was studied on peach and pineapple juice samples.

At the beginning of the test (day 0), all of the samples (three bottles for each juice flavor) were analyzed in triplicate, and all of them contained significant amounts of total Sb concentrations (0.36 ± 0.01 for peach juice and 0.50 ± 0.02 µg Sb L^−1^ for pineapple juice (*n* = 9)).

For the samples stored at 4 °C and 20 °C, no variations in total Sb concentration were observed over time up to 30 days. 

**Figure 1:**
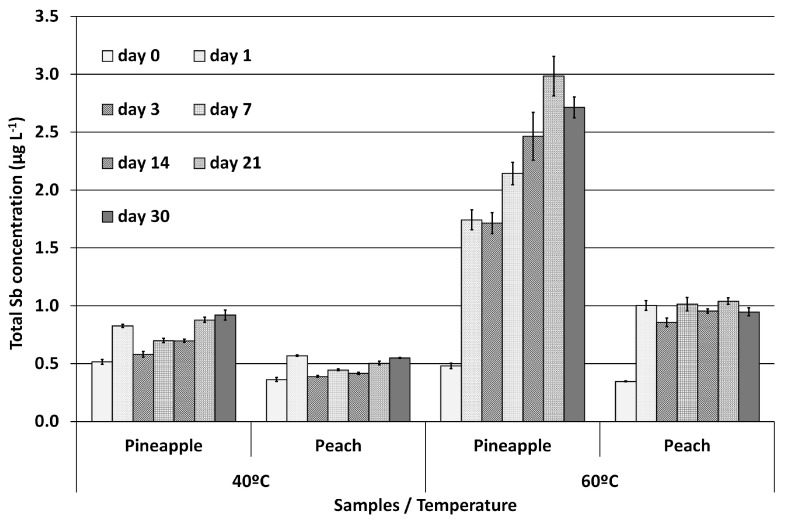
Total antimony concentration (μg·L^−1^) in peach and pineapple juice PET-stored samples at 40 °C and 60 °C (mean (*n* = 9) ± standard deviation).

Results of the experiments performed on PET stored samples at 40 °C and 60 °C are represented in Figure 1, which summarizes total Sb concentrations in juices over the month of storage. 

As speciation analysis is concerned, all the samples contained Sb(III) as the major species at any temperature during the whole experiment. The concentration levels followed the same tendency as described for total Sb analysis. Small amounts of uncomplexed Sb(V) were also observed near the limit of detection, along with some unidentifiable signals of very low intensity. Storage at 4 °C and 20 °C did not result in a significant evolution of Sb concentrations. Sb(III) concentrations in the samples stored at 4 °C and 20 °C were 0.13 ± 0.02 and 0.27 ± 0.04 µg Sb L^−1^ for peach and pineapple, respectively. Storage at 40 °C and 60 °C showed a slight increase in Sb(III) concentration up to day 30.

**Figure 2:**
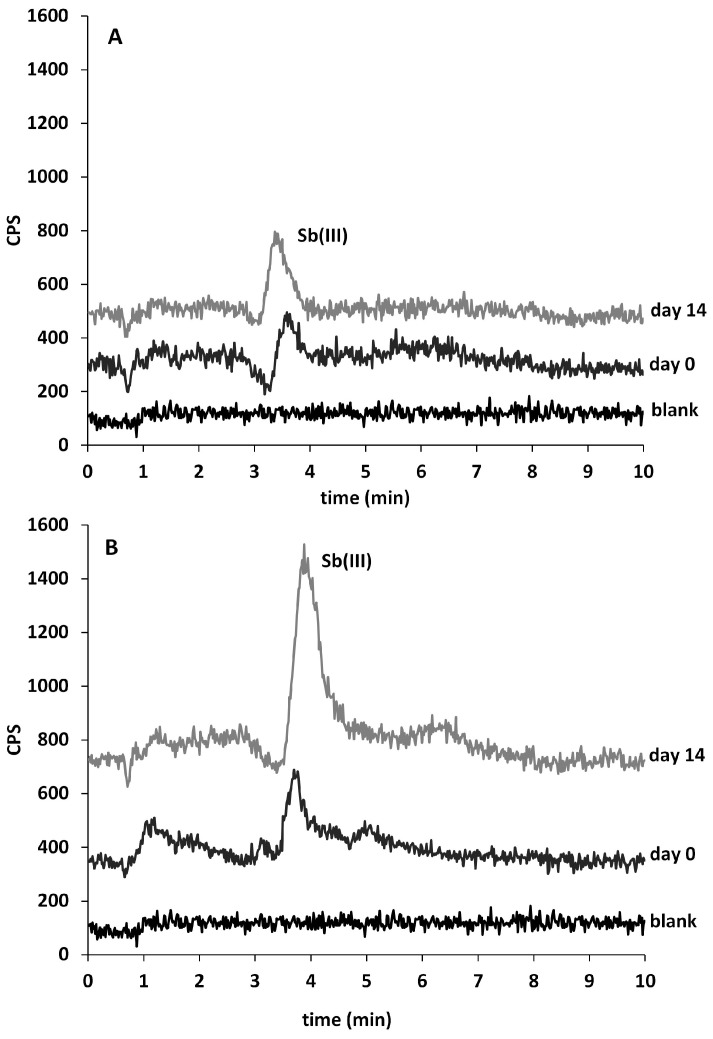
Chromatograms obtained by LC-ICP-MS from PET-stored peach juice (**A**) and PET-stored pineapple juice (**B**) at 60 °C at different days.

Sb(III) average concentrations in the samples stored at 4 °C and 20 °C were 0.13 ± 0.02 and 0.27 ± 0.04 µg Sb L^−1^ for peach and pineapple, respectively, together with the concentrations in the samples stored at 40 °C and 60 °C. It can be observed that the concentrations of Sb(III) are lower than those obtained in the total Sb determination: The peach samples reached Sb concentrations of 0.17 ± 0.01 and 0.18 ± 0.02 µg Sb L^−1^ at 40 °C and 60 °C, respectively, whereas for the pineapple samples, these values are 0.53 ± 0.04 and 1.02 ± 0.09 µg Sb L^−1^, respectively. RSD values were ≤10% in all cases. Figure 2 shows the chromatograms obtained from PET stored peach and pineapple juice at 60 °C at different times, along with a blank for comparison purposes.

### 2.4. Cross-Migration Experiments

To study the extent to which Sb migration is governed by matrix and PET characteristics, a cross-migration experiment was carried out. The concentrations obtained at the beginning of the experiment (1.5 L bulk sample) (day 0) are summarized in Table 2. Total Sb concentrations in waters were slightly lower than those obtained in juices. Regarding speciation, the results obtained were the same as those obtained in the previous migration experiments: uncomplexed Sb(V) was the only species present in the water samples, whereas in juices, Sb(III) was the predominant species, and uncomplexed Sb(V) was present under the LOQ.

**Figure 3:**
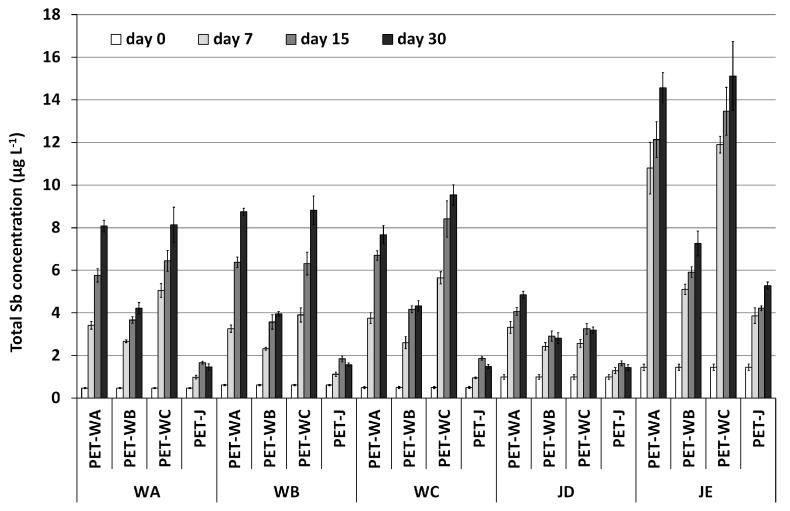
Total Sb concentrations (mean *n* = 3, standard deviation in bars) in mineral drinking waters and fruit juices samples used for the cross-migration experiment, stored at 60 °C for one month. In X-axis, the outer labels correspond to the beverage (water or juice), and the inner labels correspond to the PET bottle.

Total Sb concentrations in the reference set of samples kept at 20 °C and after 30 days of crossed storage were not different from the initial contents, whereas for samples stored at 60 °C, some changes were observed already from day 7 of storage, as can be seen in Figure 3.

**Figure 4:**
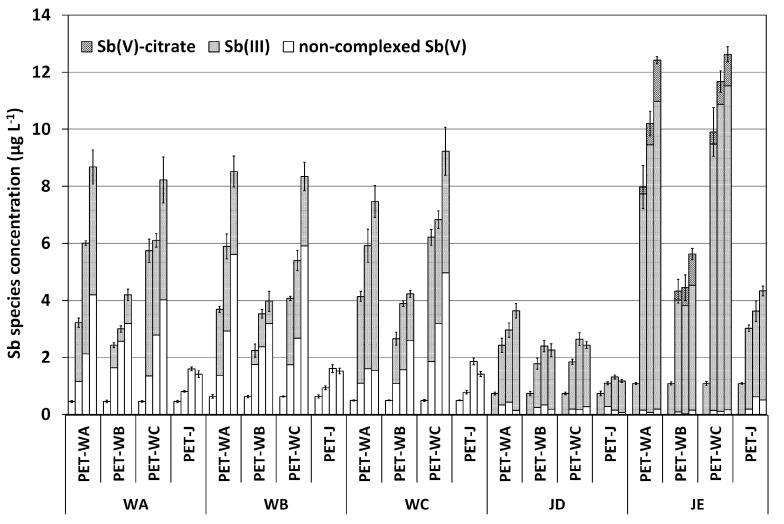
Sb species concentrations (mean *n* = 3, standard deviation in bars) in mineral drinking waters and fruit juices samples used for the cross-migration experiment, stored at 60 °C for one month.

Regarding the speciation results, the antimony species concentrations obtained in the cross-migration experiment are summarized in Figure 4. On the X-axis, the outer labels correspond to the beverage (water or juice), and the inner labels correspond to the PET-bottle. For the groups of 4 bars grouped in each inner label, each column corresponds to the species concentrations measured at day 0, 7, 15, and 30, from left to right.

Column recoveries of the sum of species calculated with respect to the total Sb concentration were between 80 and 100% for water samples and ranged between 70 and 85% for juices, which are acceptable for speciation in complex matrices.

## 3. Discussion

### 3.1. Determination of Total Sb in Juices

Independently of the juice flavor, the obtained concentrations of Sb were of the same order of magnitude in all samples and had good RSD values (≤5%). The maximum level of Sb established by the European Union in drinking water (5.0 µg L^−1^) was not exceeded in any sample.

These results are comparable with those reported in the literature, with Sb concentrations ranging from 0.28 to 1.05 µg L^−1^ in orange and lemon juice contained in PET bottles 31 and 0.02 to 1.20 µg L^−1^ in apple, plum, and sour cherry juice [36]. Sb concentrations reported in 25 PET-bottled red fruit juices were lower than 0.5 µg L^−1^ in 10 of the samples analyzed, while 7 of them were above the maximum level established by EU for drinking water (5 µg L^−1^) [30].

### 3.2. Sb Speciation in Juices: Optimisation of the Analytical Method

To start this study, an LC-ICP-MS method previously developed by the research group for water analysis was applied [15]. As it has been reported that different antimony species from those present in water may be present in juice because of the formation of complexes with the organic acids citric and malic [32,35], the separation process was assessed.

Firstly, to check the presence of antimony complexes in juice matrices, direct injection of pineapple and peach juices for high-resolution mass spectrometry (HRMS) was carried out. The mass spectra obtained showed high amounts of citric and malic acids but no Sb complexes. This is likely due to the very low concentration of antimony present. To observe the formation of Sb complexes, spiked samples were analyzed. In Sb(V)-spiked samples, the presence of the 1:1 citrate complex was detected, whereas in Sb(III)-spiked samples, the 1:2 citrate complex was observed. Moreover, the presence of malic complexes was not detected. 

**Figure 5:**
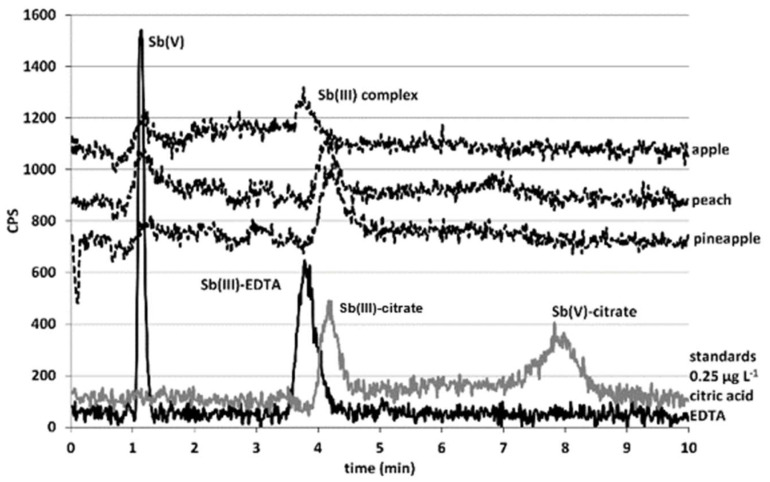
Chromatograms obtained from apple, pineapple, and juice samples together with antimony standards in mobile phase or citrate medium.

To identify the species occurring in the LC system, the analyses of individual Sb(V) and Sb(III) standard solutions of 0.25 µg L^−1^ diluted in the mobile phase (EDTA 10 mmol L^−1^) and citric acid 40 mmol L^−1^ (simulating the concentration in juice matrix) were carried out. The results of analyzing the standards, depicted in Figure 5, showed that Sb species in the mobile-phase medium have different retention times from those diluted in citrate medium. This effect is due to the formation of different complexes between antimony and ethylenediaminetetraacetic acid (EDTA) or citrate, which have different structures and consequently retention capacities. When using EDTA standards, the retention times were as follows: 1 min for Sb(V), which corresponds to uncomplexed Sb(V), and slightly less than 4 min for Sb(III), which corresponds to an Sb(III)–EDTA complex [37]. When using citrate standards, the elution order is reversed: Retention times were slightly more than 4 min for Sb(III) and approximately 8 min for Sb(V). While the pentavalent species corresponds to an antimony–citrate complex [35], it is not clear whether Sb(III) corresponds to an antimony–citrate or antimony EDTA complex, as the retention times are similar.

The analysis of the seven different-flavored PET-bottled commercial juices used in the previous section for the total Sb determination was carried out after centrifugation and filtration, as described above. Samples were analyzed with the conditions described in the Instrumentation section. As representative results, the upper part of Figure 5 depicts the chromatograms obtained for the pineapple, peach, and apple samples together with the chromatograms of the previously mentioned standards. Chromatographic peaks with the same retention times as Sb(III) standards were observed, and small amounts of uncomplexed Sb(V) were observed at 1 min.

To confirm the identity of the species observed in the juices corresponding to the complexes mentioned, spiking tests were performed. Samples were spiked with individual Sb(V) and Sb(III) standards diluted in both EDTA and citric acid. When Sb(V)-citrate was added, the 8 min peak corresponding to this standard appeared. Additionally, when the Sb(V) standard was added in EDTA media, a small peak corresponding to uncomplexed Sb(V) at a retention time of 1 min could be observed together with the peak of Sb(V)–citrate (approximately 70% of added Sb). This fact demonstrated that Sb(V) is complexed with citrate present in the juice.

Furthermore, the intensity of the main peak present in the samples (at approximately 4 min) increased when either Sb(III)–citrate or Sb(III)–EDTA was added. This behavior demonstrates that this peak corresponds to Sb(III) species, but it is still unclear whether the species observed in the chromatogram is an Sb(III)–citrate or Sb(III)–EDTA complex. However, it is suspected that this peak corresponds to an Sb(III)–EDTA complex, as it has been found in the literature that, although Sb(III)–citrate complexes are well known [31], Sb(III) complexes convert to Sb(III)–EDTA complexes on the column when EDTA is part of the mobile phase [35].

**Figure 6:**
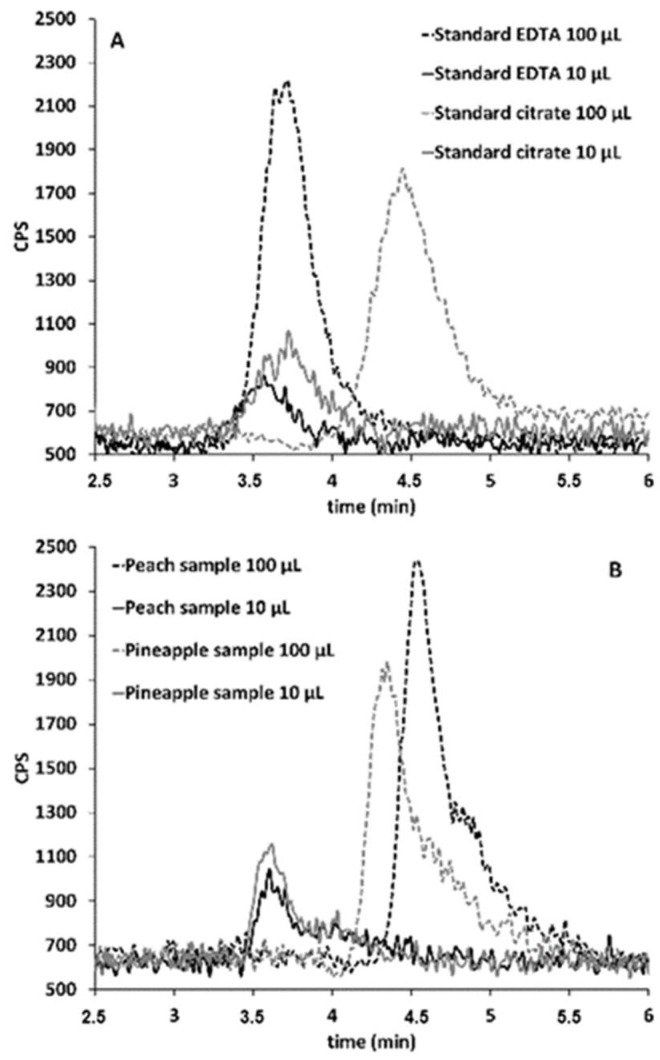
Chromatographic profiles obtained from 100 and 10 μL injections of 1 μg·L^−1^ standards of Sb(III) prepared in EDTA and citric media (**A**) and 1 μg·L^−1^ spiked peach and pineapple samples (**B**).

Therefore, to check if the Sb(III) species observed in the standards prepared with citric acid and the juice samples corresponds to Sb(III)–EDTA or to Sb(III)–citrate complexes, two different approaches were carried out. The first approach was to inject decreasing volumes of 1 µg L^−1^ Sb(III) standards prepared in EDTA and citrate media and 1 µg L^−1^ Sb(III)-spiked pineapple and peach samples: 100, 50, 25, 10, and 5 µL. In the chromatogram profiles, the retention time of the EDTA media standard was the same at each volume injected. On the other hand, the retention time of the Sb(III) peak in the citrate media standard and the samples got closer to the retention time of the Sb(III)–EDTA standard as lower volumes were injected. From an injection volume of 10 µL, the retention time was the same as the Sb(III)–EDTA standard. As an example, Figure 6 depicts the chromatographic profiles of the standards prepared with citric acid and EDTA and the spiked samples when injecting volumes of 100 and 10 µL. Thus, it can be concluded that Sb(III) species eluted with an EDTA mobile phase from the standard prepared with citric acid and also from juice samples convert to Sb(III)–EDTA complexes. At higher working volumes and due to matrix effect, the Sb(III)–EDTA complex eluted at higher retention times.

The conversion of Sb(III)–citrate species to Sb(III)–EDTA complexes was confirmed using high-resolution mass spectrometry (HRMS), by analyzing standards of Sb(III) in the presence of the stoichiometric concentration of EDTA and 10 times the stoichiometric concentration of citric acid for the correct formation of the complex [31], with the conditions explained in the Materials and Method section. 

The Sb–citrate complex ([Sb(citH_2_)_2_]^−^; C_12_H_12_O_14_Sb^−^), with an expected *m*/*z* value of 500.9260, was not detected, whereas the complex [Sb(EDTA)]^−^ (C_10_H_12_N_2_O_8_Sb^−^) was detected with a m/z value of 408.9634 with an error lower than 5 ppm. These results confirmed that trivalent forms of antimony are preferably complexed with EDTA instead of citrate in the presence of both substances in the conditions where separation is performed.

In conclusion, MS spectrometry analyses demonstrated that Sb(III) is present in samples as a 1:2 Sb(III)–citrate complex, which is converted into 1:1 Sb(III)–EDTA complex in the presence of the mobile phase during the analysis by LC-ICP-MS.

### 3.3. Sb Speciation in Juices

A dependent Student’s *t*-test for paired samples, at the 95% confidence level, showed that the total Sb content was significantly higher than Sb(III) species content. This can be attributed to the small amounts of Sb species found in the measuring extracts, which were close to the limit of quantification, where the spread of expected results is high. Column recoveries ranged from 70 to 89%, which are acceptable for speciation in complex matrices, except for the grape sample (44%).

### 3.4. Migration Experiments

For total Sb, a two-way ANOVA at the 95% confidence level for the four temperatures and the seven periods of time tested indicated that both factors have an effect on the results. 

When considering only the results obtained at 4 °C and 20 °C for all period of storage time assayed, a two-way ANOVA showed no effect of time and temperature on total Sb concentrations, and in no case did they exceed the maximum level established by the European Union for drinking water (5.0 µg L^−1^). Samples stored at 40 °C showed a slight increase in total Sb concentration over time, reaching a concentration around 0.55 ± 0.01 µg L^−1^ for peach samples and 0.92 ± 0.04 µg L^−1^ for pineapple samples at 30 days of storage. The highest Sb migration was observed in the samples stored at 60 °C, which reached a concentration of 0.95 ± 0.04 µg L^−1^ in peach and 2.71 ± 0.09 µg L^−1^ in pineapple juice, after 21 days of storage.

The values obtained throughout the migration experiment for Sb(III), the only species present at concentrations higher than the LOQ, correspond approximately to 40–65% of the total antimony concentration for pineapple juice and to 20–45% of the total antimony concentration for peach samples. Hansen and Pergantis [35] obtained similar results, finding that the major species present in lemon and orange juices was Sb(III). According to their migration results at 50 °C, the antimony increase was mainly due to Sb(III), which is consistent with the results obtained in this study.

The lack of mass balance with respect to the total Sb content could be attributed to the minor signals that correspond not only to uncomplexed Sb(V) but also to unidentified antimony species. Apart from the increase of the main peak, Sb(III), with time, it can be visually observed that the baseline from the samples is not plain compared to that obtained from the blank. These slight increases in the background signal may be attributed to different Sb(V) complexes similar to those identified by Hansen and Pergantis [32] in spiked yogurts and juices, which cannot be separated correctly with the method conditions (strong anion exchange liquid chromatography). Moreover, specifically for peach juices, the Sb(III) signal is close to the limit of quantification, where the spread of expected results can be higher.

The only information regarding the effect of temperature on Sb migration in the literature is inconclusive, as only some juice samples stored at 50 °C for 14 days showed an increase in Sb levels [35]. In the present work, similar behavior was always observed at a fixed temperature.

The results indicate that temperature is the main factor that accelerates Sb migration into fruit juice. This effect can be attributed to the increased degradation of the PET with increasing temperature, which is well known, was described by Romao and co-workers [38], and was also observed in drinking water using X-ray absorption fine-structure (XAFS) techniques by Takahashi and co-workers [2]. Moreover, this degradation was also observed visually, as the sample bottles exhibited physical deformation. However, Sb migration also appeared to be dependent on the matrix, as it was more pronounced in pineapple than in peach juice, as can be observed in Figure 1 and Figure 4. Comparing the migration data obtained in the present work about juices to those from a previous work about migration in PET-bottled mineral water [15], lower concentrations were observed for juice samples despite the expectation of higher concentration due to the high amount of salts and organic acids present in juices, which could have potentially increased Sb extractability from PET. Additional variables studied by Welle and Franz [12] can also affect the migration behavior. Total Sb concentration in PET, bottle volume and wall thickness, activation energy, and diffusion coefficients of Sb were used to propose a mathematical model for calculating migration with temperature and time. Our migration results with juices and mineral waters matched Welle’s model, showing an analogous tendency even for the high temperatures not covered in their study. 

In addition, Fan and co-workers [39] constructed kinetic curves for antimony release following the given equation:

C = C_max_·(1 − e^−bt^)

where “C” is the released antimony concentration at storage time “t” (days), “C_max_” is the maximum concentration of released antimony (µg L^−1^), and “b” is the kinetic constant (day^−1^). Values of “C_max_” and “b” can be estimated by fitting the data obtained in the equation [39]. Thus, the maximum concentration was released, and kinetic constants were calculated for the two fruit juice samples stored during 60 days at the four temperatures.

Kinetic curves at high temperatures matched the experimental values better, as evidenced by the higher R^2^ values obtained. Considerable fewer differences in C_max_ and kinetic constants were obtained throughout the temperature range, which suggests that temperature is not the only variable that affects migration in this specific case.

Furthermore, C_max_ values (0.30–2.77 μg·L^−1^) were of the same order of magnitude as the experimental maximum concentrations obtained (0.35–2.98 μg·L^−1^). This demonstrates that all samples reached the maximum allowable migrated concentration. The Sb concentration in samples from day 30 onwards, if it had been possible to analyze them, should have been equal to or of the same order of magnitude as the results obtained in the kinetic curve showing that the maximum Sb concentration was reached. Regarding the kinetic constants (b), the values are of the same order in all cases (0.011–0.775) and are in a similar range as those obtained by Fan and co-workers [39] (0.19–0.65). This means that the migration process is similar at all temperatures. It is important to note that this migration behavior is different from that observed in mineral water samples [15]. This fact may be due to the differences between matrices and the PET containers in each case.

All these facts indicate that the antimony migration behavior is governed not only by the composition of the matrix but also by the characteristics of the PET container. This is the possible explanation for the results obtained in mineral waters and juices, which are packaged in different PET containers. Consequently, cross-migration experiments were designed.

### 3.5. Cross-Migration Experiments

For the results of total Sb in the reference samples stored at 20 °C after 30 days, a Student’s *t*-test for means at 95% confidence level indicated no significant differences.

However, as expected, total antimony concentrations showed a significant increase throughout the days when stored at 60 °C. A two-way ANOVA test (95% confidence) confirmed the effect of storing time at this temperature. It can be observed that samples stored in bottles used for keeping mineral water (the first three groups of columns in Figure 3) exhibited more pronounced migration than those stored in bottles used for keeping juice (the last group of columns), as they reached higher Sb concentrations throughout the days. After 30 days of storage, mineral water samples stored in PET-WA and PET-WC bottles reached concentrations between 8 and 10 µg L^−1^, while those stored in PET-WB bottles reached a concentration of approximately 4 µg L^−1^, and those stored in PET-J bottles reached even a smaller concentration (around 2 µg L^−1^).

Peach juice samples stored in PET-WA bottles reach a concentration of around 5 µg L^−1^, while those stored in PET-WB and PET-WC bottles reach around 3 µg L^−1^, and those stored in PET-J bottles reach less than 2 µg L^−1^. Similarly, pineapple juice stored in PET-WA and PET-WC bottles reach approximately 15 µg L^−1^, while those stored in PET-WB bottles reach around 7 µg L^−1^, and those stored in PET-J bottle reach less than 5 µg L^−1^.

Based on these results, it can be concluded that antimony migration depends on both the type of PET container and the sample matrix. Samples stored in PET-WA and PET-WC bottles exhibited the highest migration potential, followed by the PET-WB bottles, and then the PET-J bottles. Additionally, for each PET container, antimony migration was also dependent on the sample matrix. Antimony concentrations after 30 days of storage were lowest in peach juice samples, higher in mineral waters, and highest in pineapple juice samples.

Regarding antimony speciation (Figure 4), only non-complexed Sb(V) was present in water samples at the beginning of the experiment. After storage time, the concentration of non-complexed Sb(V) continued to increase, and Sb(III) species appeared in waters stored in all PET-bottle types. Sb(III) species were not observed in waters stored in the PET-J type.

For juice samples, non-complexed Sb(V) below the limit of quantification and Sb(III) were the only species present at the beginning of the experiment. After the storage at 60 °C, Sb(III) concentrations increased and remained the main species present in the samples. Non-complexed Sb(V) concentrations also increased, reaching values slightly above the limit of quantification. Additionally, in pineapple juice samples stored in PET water bottles, Sb(V)–citrate species appeared, reaching values between 1 and 1.5 µg L^−1^ at the end of the experiment.

## 4. Materials and Methods

### 4.1. Instrumentation

For total Sb determination by inductively coupled plasma–mass spectrometry (ICP-MS), an Agilent 7500 ce model with a reaction cell and Burgener Ari Mist HP nebulizer was used. More information about the ICP-MS system’s operating conditions is summarized in Table 3.

For Sb species determination, a quaternary pump Agilent 1200 (Waldbronn, Germany) equipped with an auto sampler and a Hamilton PRP-X100 (Reno, NV, USA) anion exchange column (125 × 4.1 mm, 10 µm particle size, USA) were used. This HPLC system was coupled to the ICP-MS, which operated under the following conditions: room temperature, 10 mM ethylenediaminetetraacetic acid (EDTA) at pH 4.0 with 0.5% MeOH as the mobile phase; 100 µL injection volume and 1.5 mL min^−1^ flow rate.

To characterize the structure of antimony complexes by high-resolution mass spectrometry (HRMS), a Thermo Fisher Scientific Q-Exactive Orbitrap (Madrid, Spain) equipped with a thermally assisted electrospray ionization source was used. The instrument was operated in negative mode with the following parameters: ESI voltage −2.5 kV; vaporizer and capillary temperatures 320 °C; sheath gas, auxiliary gas and sweep gas flow rates 40, 10, and 2 arbitrary units, respectively; and tube lens voltage 50 V. The mass spectrometers were operated in profile mode, with a scan range of *m*/*z* 100–1000 and a resolving power of 70,000 full width half maximum (FWHM) at *m*/*z* 200. An automatic gain control setting of 3 × 10^6^ with a maximum injection time of 200 ms was used.

### 4.2. Reagents and Standards

Ultrapure water with conductivity of 5–15 M Ω cm^−1^ (Millipore, Bedford, MA, USA) was used for making up volume standards and reagents.

Standard solutions of 1000 mg L^−1^ Sb(III) and Sb(V) were prepared from C_8_H_4_K_2_O_12_Sb_2_ xH_2_O (Fluka, Neu-Ulm, Germany) and KSb(OH)_6_ (Riedel de-Haën, Seelze, Germany), respectively. A certified standard solution of 1000 ± 4 mg L^−1^ antimony, prepared from 99.999% “purum” metallic Sb, dissolved and stabilized in high-purity acids (5% nitric acid (HNO_3_) and 0.1% hydrofluoric acid (HF)), was used to standardize both standards. All standard solutions were kept refrigerated in high-density polyethylene bottles. The media of the daily working standard solutions were diluted acid and mobile phase for total and speciation Sb analysis, respectively.

A 10 mM EDTA (Panreac, Barcelona, Spain) solution adjusted to pH 4 with diluted ammonia (Panreac) was filtered daily through a 0.45 μm filter (Millipore type HA) for use as the mobile phase.

LC/MS grade methanol and water (Fluka, Madrid, Spain) were used to prepare the solutions for mass spectrometry analysis.

### 4.3. Selection of Samples

To characterize juices in preliminary experiments, seven commercial juices from the same brand were purchased from a local shop in order to cover the different flavors typically consumed in Spain and bottled in PET: apple, grape, pear, pineapple, peach, orange, and tomato. Juices were centrifuged and filtered through 0.45 μm, according to the procedure described by Welna and Szymczycha [36]. Then, 2 mL of each of the filtered juices was taken and diluted. Total and speciation analysis were performed by ICP-MS and LC-ICP-MS, respectively, as quantification techniques.

Two fruit juices, pineapple and peach, were selected for migration studies to study the influence of storage time and temperature on the Sb concentration in the juice. These juices are commonly consumed in Spain and were commercially available in suitable size batches.

For the cross-migration experiment, the same pineapple and peach juices were used together with three low-mineralized PET-bottled waters from different brands.

### 4.4. Migration and Cross-Migration Experiments

Three bottles of each flavor were stored at 4 °C, 20 °C, 40 °C, and 60 °C. Speciation and total antimony were analyzed in triplicate on seven occasions: before storage and after 1, 3, 7, 14, 21, and 30 days of storage. Longer storage was not carried out because, after one month of storage, juices at 20 °C appeared spoiled, while juices at 40 and 60 °C had darkened and become almost black, with a foul smell. Thus, because the juices were already decomposed after one month of storage, there was no point in continuing to analyze them for a longer time.

For the cross-migration experiment, three low-mineralized drinking waters (WA, WB, and WC) from different brands were selected based on their different PET container (shape, color, and thickness), along with peach and pineapple fruit juices (JD and JE, respectively) from the same brand. PET containers for juices had a different shape, were thinner than water bottles, and were colorless. For this experiment, five 0.33 L containers of each PET type were used (PET-WA, PET-WB, PET-WC, and PET-J). The contents of each container were discarded, and bottles were cleaned three times with double deionized water and dried at room temperature. Subsequently, each set of containers (e.g., PET-WA) was filled with all studied matrices (WA, WB, WC, JD, and JE), meaning that each matrix was placed inside all types of PET containers studied. For filling, a 1.5 L container of the studied matrices (waters and juices) was used. The content of the 1.5 L containers was also characterized in terms of total and speciation Sb content.

After filling, the bottles were stored at 60 °C for 30 days. Total Sb and speciation were analyzed in triplicate after 7, 15, and 30 days of storage. For reference, another set of bottles prepared as previously described was kept at 20 °C, and total Sb content was measured after 30 days of storage.

### 4.5. Determination of Antimony by ICP-MS

#### 4.5.1. Total Analysis

A 2 mL aliquot of the sub-sample was diluted to 4 mL in HNO_3_ 1 mol L^−1^. The samples were introduced into the ICP-MS system under the conditions described in the Instrumentation section. The samples were quantified using external calibration curves with total Sb standards from 0.1 to 7.5 µg L^−1^. Each sample was analyzed in triplicate. For quality control purposes, rhodium was added to the nebulizer chamber to detect potential signal drifts during the working session. Additionally, the calibration curves standards were run before and after each sample series. Multi-elemental quality control standard solutions at two concentrations were measured at the end of the sequence to ensure stable instrument sensitivity.

#### 4.5.2. Speciation Analysis

A 100 μL aliquot of sub-sample was directly injected into the LC system under the conditions described in the Instrumentation section. External calibration was used for quantification purposes using Sb(III) and Sb(V) solutions. After identifying the Sb species in the extracts by comparing their retention times to those of the standards, quantification was performed in the working range 0.05 to 2.5 µg L^−1^ for each species. The instrument stability during the working sessions was checked by measuring two Sb quality control standard solutions at the end of the sequence, and the calibrating standards were also run before and after each series of analysis. Data were acquired using ICP-MS Chemstation software (version C.01.00), and the peak areas were integrated using ICP-MS Chromatographic Data Analysis software (version C.01.00).

The same separation and measuring conditions proposed for the analysis of Sb in waters by LC-ICP-MS [15] can be also applied to the analysis of Sb species in the proposed juices, with a 10 min analysis time. The limits of detection (LOD) for the method are 0.01 and 0.03 μg·L^−1^ for Sb(V) and Sb(III), respectively, and the limits of quantification (LOQ) are 0.03 and 0.09 μg·L^−1^ for Sb(V) and Sb(III), respectively.

For quantification, standards prepared in the mobile-phase and citrate media are necessary because the presence of non-complexed Sb(V) and Sb(V)–citrate can be only quantified using the EDTA-media curve and citrate media curve, respectively. The trivalent species can be quantified with both Sb(III) calibration curves because the slopes obtained from the curves prepared with Sb(III)–EDTA and Sb(III)–citrate standards are not significantly different.

#### 4.5.3. Structure Characterization by HRMS

A 2 μL aliquot of standard solution was filtered through a 0.22 μm filter and directly injected into the mass spectrometer operating under the conditions described in the Instrumentation section. Thermo Xcalibur Qual Browser software (version 3.1.) was used for instrument control and data acquisition.

### 4.6. Statistical Analysis

Students’ *t*-test for comparing means, paired *t*-test for comparing series of data, and two-way ANOVA to ascertain the effect of factors affecting migration were carried out using Microsoft^®^ Excel^®^ 2016.

## 5. Conclusions

From the obtained results, it can be concluded that temperature is the main kinetic factor affecting antimony migration from PET to juice, as warmer sample storage leads to higher antimony migration from the bottle into the contents. Antimony (III) was the main species observed throughout the migration experiment.

Cross-migration experiments showed that antimony migration behavior can only be explained by considering both matrix composition (content) and PET bottle characteristics (container). The strategy used in this paper to study antimony migration proved to be a useful approach for assessing the possible role of the matrix and the PET type simultaneously.

The crucial role of the matrix composition in Sb migration from PET to food proven in this work should also be addressed in future updates to the present European legislation describing migration assays using food simulants such as water, acetic acid, and nitric acid. When complexing agents are present in the food matrices, as in the case of juice, simulants may underestimate the migrated amounts. Further work should ascertain the correlation between citric acid (and pH) and Sb concentration in the solvent in order to propose universal food simulants that improve food safety.

## Figures and Tables

**Table 1:** Total antimony concentration (mean ± standard deviation) in juices determined by ICP-MS using as pre-treatment a sample centrifugation (*n* = 3, from different replicates of every sample).

Juice	Concentration (µg L^−1^)	RSD (%)
Apple	0.343 ± 0.002	0.7
Grape	0.937 ± 0.018	1.9
Pear	0.522 ± 0.008	1.6
Pineapple	0.338 ± 0.002	0.5
Peach	0.273 ± 0.009	3.2
Orange	0.364 ± 0.005	1.3
Tomato	0.166 ± 0.007	4.2

**Table 2:** Antimony concentrations obtained in water samples (WA, WB, and WC), and juice samples (pineapple, JD; peach, JE) at the beginning of the experiment (day 0). Results are expressed as mean Sb value ± standard deviation (*n* = 3), and the relative standard deviations are expressed in % in parentheses.

Sample	Total Sb (µg L^−1^)	Sb(V) (µg L^−1^)	Sb (III) (µg L^−1^)
WA	0.47 ± 0.03 (6.9%)	0.46 ± 0.02 (5.0%)	<LOD
WB	0.61 ± 0.04 (6.2%)	0.63 ± 0.06 (8.9%)	<LOD
WC	0.49 ± 0.05 (9.8%)	0.50 ± 0.05 (9.2%)	<LOD
JD	0.99 ± 0.11 (10.7%)	<LOQ	0.74 ± 0.04 (5.7%)
JE	1.45 ± 0.15 (10.1%)	<LOQ	1.09 ± 0.03 (3.0%)

**Table 3:** Operating conditions of the ICP-MS system.

ICP-MS Parameters	
RF power	1550 W
RF matching	1.66 V
Peristaltic pump speed	0.1 rps
Stabilization delay	30 s
Sampler and skimmer cones	Nickel
Nebulizer	BURGENER Ari Mist HP
Number of replicates	3
Spray chamber (type and temperature)	Scott-type and 15 °C
Carrier gas flow, Ar	0.75 L min^−1^
Make up gas flow, Ar	0.33 L min^−1^
Sampling depth	7.5 mm
Cell exit	−36 V
Quadrupole/Octopole bias difference	3 V
Mass to charge ratio	*m*/*z* 121 (^121^Sb)

## Data Availability

Not applicable.
